# Penal Code State of New York

**Published:** 1907-09

**Authors:** 


### PENAL CODE STATE OF NEW YORK.

Section 401 of the Penal Code has been amended so as to read as follows:

Any person, who, putting up any drug, medicine or food or preparation used in medical practice, or making up any prescription, or filling any order for drugs medicines, food or preparation puts any untrue label, stamp or other designation of the contents of the box, bottle to other package containing a drug, medicine, food or preparation used in medical practice, or substitutes or dispenses a different article for or in lieu of any article prescribed, ordered or demanded, or puts up a greater or less quantity of any ingredient specified in any such prescription, order or demand than that prescribed, ordered or demanded, or otherwise deviates from the terms of the prescription, order or demand by substituting one drug for another, is guilty of a misdemeanor; provided, however, that except in the case of physicians' prescriptions, nothing herein contained shall be deemed or construed to prevent or impair or in any matter affect the right of the apothecary, druggist, pharmacist or other person to recommend the purchase of an article other than that demanded, ordered or required, but of a similar nature, or to sell such other article in place or in lieu of the article ordered, required or demanded, with the knowledge and consent of the purchaser. Upon a second conviction for a violation of this section the offender must be sentenced to imprisonment, for a term of not less than ten days nor more than one year, and to the payment of a fine of not less than ten or more than five hundred dollars. The third conviction of a violation of any of the provisions of this section, in addition to rendering the offender liable to the penalty prescribed by law for a misdemeanor, shall forfeit any right which he may possess under the law of this state at the time of such conviction, to engage as proprietor, agent, employee or otherwise, in the business of an apothecary, pharmacist or druggist, or to compound, prepare or dispense prescriptions or orders for drugs medicines or foods or preparations used in medical practice; and the offender shall be by reason of such conviction disqualified from engaging in any such business as proprietor, agent, employee or otherwise, or compounding, preparing or dispensing medical prescriptions or orders or drugs, medicines, or foods or preparations used in the medical practice.

Section 402· This act shall not affect or inpair any liability, penalty or punishment under the provisions of section four hundred and one as the same existed prior to the time this act takes effect, but the same may be enforced, prosecuted or inflicted as fully and to the same extent as though this act had not been passed; and all actions civil or criminal instituted under or by virtue of said section as the same existed prior to the passage of this act, and pending immediately prior to the taking effect hereof, may be prosecuted and defended to final action in the same manner as though this act had not been pased.

Section 403. This act shall take effect September first, 1907.

